# Case Report: Ectopic pulmonary embolism as a complication of bronchial artery embolization

**DOI:** 10.3389/fcvm.2024.1456360

**Published:** 2024-09-02

**Authors:** Min Liu, Jixiang Liu, Shen Chen, Xiaoyan Gao, Jinnan Zhong, Lu Sun, Fajiu Li, Chenghong Li

**Affiliations:** ^1^ Department of Pulmonary and Critical Care Medicine, Affiliated Hospital of Jianghan University, Wuhan, China; ^2^ National Center for Respiratory Medicine, State Key Laboratory of Respiratory Health and Multimorbidity, National Clinical Research Center for Respiratory Diseases, Institute of Respiratory Medicine, Chinese Academy of Medical Sciences, Department of Pulmonary and Critical Care Medicine, Center of Respiratory Medicine, China-Japan Friendship Hospital, Beijing, China; ^3^ Institute of Pulmonary Vascular Diseases, Jianghan University, Wuhan, China

**Keywords:** case report, hemoptysis, bronchial artery embolization, atypical bronchopulmonary fistula, ectopic pulmonary embolization

## Abstract

Bronchial artery embolization (BAE) is currently the first-line treatment for massive hemoptysis. Previous studies have proven its safety and efficacy, with mild, transient, and reversible complications. This case described a patient with congenital multiple bronchopulmonary fistulas who underwent BAE due to massive hemoptysis. However, due to an overlooked and misdiagnosed atypical fistula, the patient experienced an ectopic pulmonary embolism and subsequently secondary pulmonary infarction. He eventually exhibited a full postoperative recovery following percutaneous catheter-directed embolectomy. This case revealed a type of occult fistula masked by multiple bronchial artery branches, which may be a potential risk factor for an ectopic pulmonary embolism during BAE. We propose that it is crucial to identify abnormal anastomosis, especially atypical fistula, and select appropriate embolization materials during BAE.

## Introduction

Bronchial artery embolization (BAE) is the primary treatment for recurrent hemoptysis unresponsive to drug therapy, particularly in patients with progressive hemoptysis (1, 2). Despite its safety and efficacy, BAE carries an inherent risk of complications. For example, ectopic embolism is one of the rare and serious complications that arise as a result of vascular malformations of the pulmonary and bronchial arteries, such as fistulas. These include bronchial artery-pulmonary artery fistula, bronchial artery-pulmonary venous fistula, pulmonary arterio-venous fistula, and other abnormal communications between systematic artery and pulmonary vessels, which can be further divided into congenital and acquired fistula (3–6).

Herein, we report a patient with multiple congenital bronchopulmonary artery fistulas complicated by an ectopic pulmonary embolism during BAE. In this case, a type of occult fistula masked by multiple bronchial artery branches was described. It confirmed that materials injected into the bronchial circulation could indeed reach the pulmonary circulation during BAE.

## Case presentation

A 59-year-old man was admitted due to acute hemoptysis, with an estimated blood loss of 100–200 ml/day. The patient had a history of hypertension for more than 5 years and was treated with amlodipine (5 mg once daily). He had no prior chronic lung disease and was a non-smoker, without alcohol consumption, illicit drug use, and any home or occupational exposures. On admission, a physical examination revealed a body temperature of 36.4℃, blood pressure of 135/80 mmHg, heart rate of 80 beats per minute, respiratory rate of 18 breaths per minute, and oxygen saturation of 93% on room air. A chest CT scan showed scattered ground-glass opacities in the right middle lobe. His coagulation profile, renal function, and liver function were within normal limits. Initial treatment with Hemocoagulase Bothrops Atrox (2U intravenously twice daily) and vitamin K1 (10 mg intravenous drip once daily) was administered without yielding satisfactory effects. Subsequently, emergency BAE was performed due to sudden massive hemoptysis.

**Figure 1:**
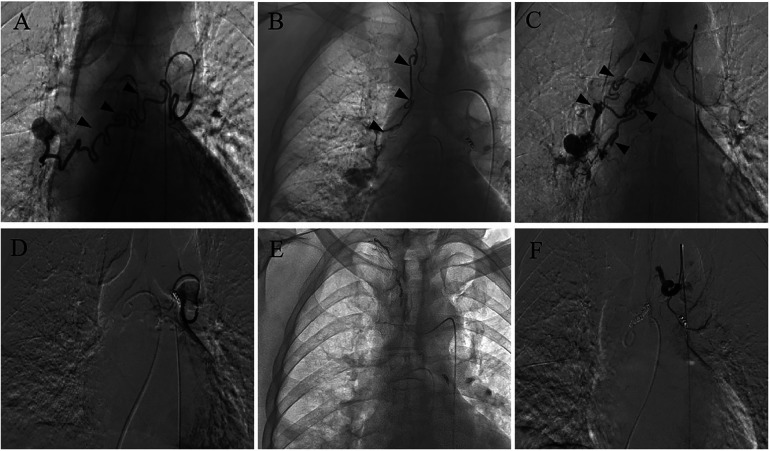
Bronchial arteriography before and after BAE. **(A)** A fistula between the right bronchial artery and pulmonary artery. **(B)** A fistula between the subclavian artery and pulmonary artery. **(C)** An atypical fistula, originating from the arcus aortae, between the right bronchial artery and pulmonary artery, featuring multiple tortuous and dilated branches with distal vascular beds. **(D**–**F)** The coil successfully embolized the target vessel and the bronchial artery-pulmonary artery fistula was occluded after BAE.

**Figure 2:**
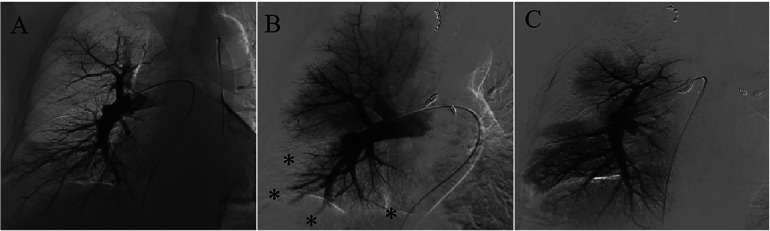
Pulmonary angiography before and after BAE. **(A)** There was no filling defect in the pulmonary artery before BAE. **(B)** Filling defects in the right subsegmental pulmonary artery after BAE were confirmed. **(C)** Restoration of pulmonary perfusion after percutaneous catheter-directed embolectomy.

Bronchial angiography identified multiple anastomoses between the hypertrophic bronchial arteries and the right subsegmental pulmonary artery (Figure 1), without filling defects in the pulmonary artery, as confirmed by pulmonary angiography (Figure 2A). Two typical fistulas, originating from the right bronchial artery (Figure 1A) and the subclavian artery (Figure 1B), were identified and successfully embolized with coils (Figures 1D,E). Another atypical fistula, arising from the aortic arch, between the right bronchial artery and the pulmonary artery was initially misdiagnosed due to tortuosity, dilation, and hyperplasia at the vessel end (Figure 1C). Therefore, 500–700 µm polyvinyl alcohol (PVA) particles were used to embolize the terminal branches of the aberrant bronchial artery. However, during the procedure, it was noted that some embolic particles seemed to disappear, resulting in an ineffective embolization of the target vessels. Coils were subsequently used to embolize the culprit bronchial arteries (Figure 1F). Considering the possibility of an ectopic embolism due to particle translocation, selective pulmonary angiography confirmed filling defects in the right lower lobe (Figure 2B). Given the non-thrombotic embolism and the potential risk for recurrence of hemoptysis, anticoagulation and antiplatelet therapies were not prescribed. In addition, the patient had no special discomfort and declined attempts to remove the particles via endovascular intervention. He was discharged with alleviated hemoptysis 3 days later.

**Figure 3:**
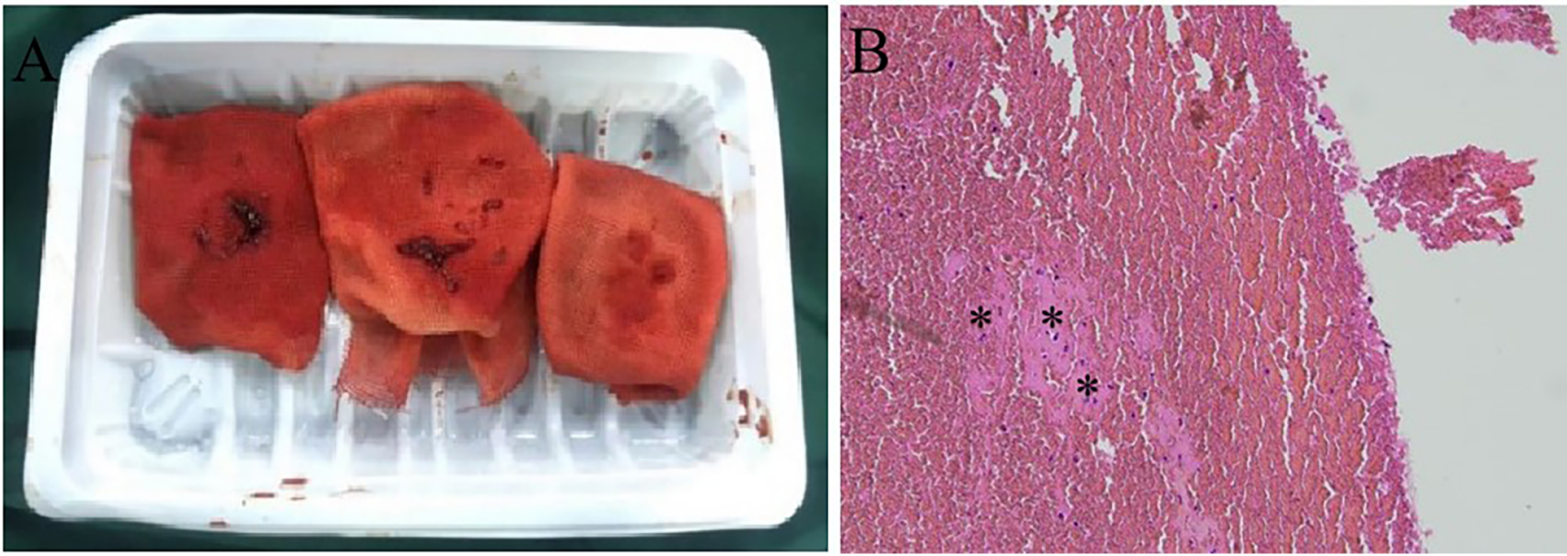
Percutaneous catheter-directed embolectomy. **(A)** The granular beaded material and thrombus mixture was aspirated in surgery. **(B)** Histological examination confirmed the presence of a mixed thrombus with scattered refractive, round-like, or patchy particles.

However, 2 weeks later, the patient was readmitted to the emergency department due to recurrent hemoptysis. Ultrasonography demonstrated no deep vein thrombosis. Computed tomography pulmonary angiography revealed a pulmonary embolism accompanied by secondary pulmonary infarction. He was administered therapeutic low-molecular-weight heparin and subsequently underwent a percutaneous catheter-directed embolectomy. Postoperative pulmonary angiography demonstrated restoration of pulmonary perfusion (Figure 2C). The granular beaded material and thrombus mixture was removed (Figure 3A). Histological examination confirmed a mixed thrombus with scattered refractive, round-like, or patchy particles (Figure 3B). It was thought to be *in situ* thrombosis in the pulmonary artery secondary to translocated particles during the interventional procedure. The following day, hemoptysis was stopped with remarkable relief of his dyspnea. He was discharged and switched to oral rivaroxaban for 3 months without thrombotic recurrence. There were no recurrences of episodic dyspnea or hemoptysis during the 12-month follow-up.

## Discussion

We report a case of ectopic pulmonary embolism following BAE. To the best of our knowledge, this is the first report that a particulate embolic agent caused an ectopic pulmonary embolism and secondary pulmonary infarction during the procedure. According to the literature, the overall complication rate of BAE is 13.4%, including 0.2% with major complications (7). Improper technique has resulted in complications such as hematoma at the puncture site, pseudoaneurysm, stress hypertension, bronchial artery, or aortic dissection (8). Post-embolization syndrome includes fever, chest tightness, chest pain, cough, dysphagia, and hiccups (9). Rare complications encompass tracheal fistula, broncho-esophageal fistula, diaphragmatic paralysis, myocardial infarction, micro-infarctions in renal and splenic tissues, ischemic bowel disease, systemic infarctions, and localized skin necrosis (10–15). Notably, neurological complications are one of the most severe complications of BAE (16, 17). These complications manifest as transient dysfunctions or irreversible impairments (18, 19).

Post-embolization syndrome is one of the most common complications. This may stem from local irritation due to the embolic material and ischemic tissue injury from the embolization of the target vessel. One of the most severe complications of BAE is an ectopic embolism due to non-targeted embolization. Inadequate hyperselective embolization, challenges in super-selection, or suboptimal angiography can result in misembolization. Backflow of embolic material and plaque shedding from the vascular wall may also lead to an ectopic embolism. In addition, anastomosis, especially fistula between the bronchial arteries and other vessels, is the most common cause of ectopic embolization. Embolic agents could pass through anastomotic branches into the pulmonary or systemic circulation, resulting in a pulmonary embolism or even multiple organ micro-infarctions (20, 21). In our case, we revealed two typical bronchopulmonary artery fistulas and successfully embolized these with coils. Another atypical fistula, featuring multiple tortuous and dilated branches, was overlooked and misdiagnosed. An ectopic pulmonary embolism still occurred despite PVA particles with a diameter of 500–700 μm being used. As there were no prior pulmonary diseases, this case is considered a congenital bronchopulmonary artery fistula. Compared to the secondary compensatory collateral circulation, this fistula had a wider diameter, facilitating the passage of embolic particles. This case indicates that it is crucial to identify pulmonary vessel fistulas during BAE. An occult fistula masked by multiple tortuous and dilated bronchial arterial branches may be a potential risk factor for an ectopic pulmonary embolism during BAE. Physicians should be alert to the formation of fistulas during the operation. Digital subtraction angiography (DSA) is the gold standard for the diagnosis of fistulas. In suspected cases, targeted multi-angle angiography is necessary to identify these occult atypical fistulas.

Normal communication exists between the systemic and pulmonary circulations. Over 70 years ago, studies reported that substances injected into the bronchial circulation could reach the pulmonary circulation in lobectomy specimens from patients with bronchiectasis (22). Robbins et al. described a patient with chronic thromboembolic pulmonary disease who underwent BAE for hemoptysis (20). Eventually, a large amount of material composed of a thrombus and eosinophilic microspheres was found in the tissues dissected by endarterectomy. As no anastomoses between the bronchial arteries and other vessels were observed during surgery, almost all the mechanisms of ectopic embolism after BAE were suspected. In this instance, particles were found to have been translocated into the pulmonary artery via a congenital bronchopulmonary artery fistula, causing *in situ* thrombosis and pulmonary infarction, through imaging and pathological examination. This case corroborates previous studies, demonstrating that materials injected into the bronchial circulation could indeed reach the pulmonary circulation.

Currently, there are no evidence-based guidelines for treating complications following BAE. Most symptoms are transient and reversible, resolving themselves with symptomatic treatment. Although this patient experienced no immediate notable discomfort, he eventually required percutaneous pulmonary thrombectomy due to the ectopic pulmonary embolism resulting in secondary pulmonary infarction. Given the limited experience of this complication, the optimal treatment strategy remains unclear. This case demonstrates that vascular intervention may be necessary for patients with a severe ectopic pulmonary embolism. Further research is essential to develop strategies for preventing and managing this potentially severe complication.

In conclusion, embolization materials injected into the bronchial circulation were vulnerable to translocating into the pulmonary artery. It is crucial to identify abnormal communications, particularly occult atypical fistulas, between the bronchial and pulmonary circulations to prevent ectopic pulmonary embolization during BAE. Although most postoperative complications of BAE are minor, vigilance is still essential due to the potential risk of serious complications.

## Data Availability

The original contributions presented in the study are included in the article, further inquiries can be directed to the corresponding authors.
